# Improvement of Cancer Immunotherapy by Combining Molecular Targeted Therapy

**DOI:** 10.3389/fonc.2013.00136

**Published:** 2013-05-28

**Authors:** Yutaka Kawakami, Tomonori Yaguchi, Hidetoshi Sumimoto, Chie Kudo-Saito, Tomoko Iwata-Kajihara, Shoko Nakamura, Takahiro Tsujikawa, Jeong Hoon Park, Boryana K. Popivanova, Junichiro Miyazaki, Naoshi Kawamura

**Affiliations:** ^1^Division of Cellular Signaling, Institute for Advanced Medical Research, Keio University School of Medicine, Tokyo, Japan

**Keywords:** immunotherapy, immunosuppression, MAPK, STAT3, β-catenin

## Abstract

In human cancer cells, a constitutive activation of MAPK, STAT3, β-catenin, and various other signaling pathways triggers multiple immunosuppressive cascades. These cascades result in the production of immunosuppressive molecules (e.g., TGF-β, IL-10, IL-6, VEGF, and CCL2) and induction of immunosuppressive immune cells (e.g., regulatory T cells, tolerogenic dendritic cells, and myeloid-derived suppressor cells). Consequently, immunosuppressive conditions are formed in tumor-associated microenvironments, including the tumor and sentinel lymph nodes. Some of these cancer-derived cytokines and chemokines impair immune cells and render them immunosuppressive via the activation of signaling molecules, such as STAT3, in the immune cells. Thus, administration of signal inhibitors may inhibit the multiple immunosuppressive cascades by acting simultaneously on both cancer and immune cells at the key regulatory points in the cancer-immune network. Since common signaling pathways are involved in manifestation of several hallmarks of cancer, including cancer cell proliferation/survival, invasion/metastasis, and immunosuppression, targeting these shared signaling pathways in combination with immunotherapy may be a promising strategy for cancer treatment.

## Introduction

By the time cancer cells are detected clinically, they have already evaded the immune-defense system (Robert et al., 2011). During their long development process, such cancer cells have lost highly immunogenic tumor antigens and acquired immunoresistant and immunosuppressive properties through various mechanisms (Yaguchi et al., 2011). Consequently, elimination of cancer cells by immunological strategies may not be easy. However, it has been revealed that the tumor antigens expressed by cancer cells are qualitatively or quantitatively different form the normal counterpart, and that cancer cells can be eliminated by T cells using various immune-interventions in some patients. We have previously identified human tumor antigens recognized by T cells (Kawakami et al., 1994a,b), and attempted to develop various antigen-specific immunotherapies (Rosenberg et al., 1998). For instance, the administration of gp100 melanoma antigen peptide vaccine along with IL-2 resulted in 16% objective response with 9% CR in the recent multicenter randomized trial (Schwartzentruber et al., 2011). Furthermore, adoptive immunotherapy using cultured melanoma-specific T cells following lymphomyeloablative treatment, which depletes various immunosuppressive cells and induces homeostatic proliferation of administered T cells, resulted in more than 70% objective response with about 20% durable CR in advanced melanoma patients with multiple metastases (Rosenberg et al., 2011). These observations indicate that active immunization may be further improved by various immune-interventions.

## Development of Effective Immunotherapy by Comprehensive Regulation of Anti-Tumor Immune Network

**Figure 1:**
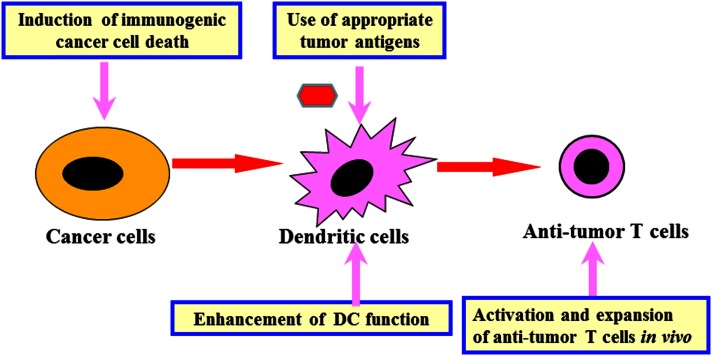
**Development of effective immunotherapy by comprehensive regulation of anti-tumor immune network**. Comprehensive regulation of anti-tumor immune network, including induction of immunogenic cancer cell death, use of appropriate tumor antigens, enhancement of DC function, activation and expansion of anti-tumor T cells *as well as* reversal of cancer-induced immunosuppression (Figure 2) is important for development of effective cancer immunotherapy.

Analysis of mouse tumor models and human clinical trials using the identified tumor antigens revealed that following key points need to be addressed in order to regulate the anti-tumor immune network and develop effective immunotherapy (Figure 1) (Kawakami et al., 2004). (1) *Identification of appropriate tumor antigens for immunotherapy*: the ideal antigens should have tumor-specific expression and they should be involved in cancer cell proliferation/survival. They must also be expressed in cancer initiating cells. We have identified human glioma antigen SOX6, which is expressed in glioma stem-like cells. SOX6 is involved in cancer proliferation and is recognized by T cells (Ueda et al., 2004, 2010). Sox6-DNA vaccination was able to inhibit growth of murine glioma in a therapeutic setting (Ueda et al., 2008). (2) *Development of in situ tumor destruction methods to induce immunogenic cancer cell death:* break down of tumor releases endogenous tumor antigens and subsequently induces anti-tumor immune response (*Immunogenic cancer cell death)*. This may be achieved possibly by using chemotherapy, molecular targeted drugs, anti-tumor antibody, irradiation, cryoablation, radiofrequency ablation, or oncolytic viruses. (3) *Development of methods to enhance dendritic cell (DC) functions:* the methods include augmentation of antigen uptake, cross presentation, and T cell stimulation by using adjuvants, cytokines, or agonistic antibodies. We have previously developed several protocols for combined immunotherapy of *in situ* tumor destruction and subsequent DC activation. An example of this is the use of oncolytic HSV, which is capable of both direct tumor destruction and DC stimulation. Intratumoral administration of HSV not only inhibited the treated tumor but also suppressed untreated tumors at remote sites via induction of systemic anti-tumor T cells (Toda et al., 2002). Another protocol involves a combination of tumor cryoablation and subsequent intratumoral administration of DCs pretreated with TLR2-stimulating BCG-CWS (Mycobacterium bovis Bacillus Calmette-Guérin cell wall skeleton). This protocol induced T cell responses to multiple endogenous tumor antigens and suppressed growth of untreated remote tumors as well (Udagawa et al., 2006). (4) *Development of methods to activate and expand anti-tumor T cells in vivo:* this may be achieved possibly by immunization with tumor antigens, administration of cytokines, or agonistic antibodies against co-stimulatory molecules on T cells, or transfer of cultured anti-tumor T cells. We are currently attempting to use tumor-specific T cells cultured *in vitro* to treat patients with melanoma. (5) *Development of methods to reverse immunosuppression*: Various immunomodulating reagents are being studied to evaluate their efficacy in recovering immunosuppressive condition in cancer patients. These reagents include antibodies (e.g., anti-CTLA-4, anti-PD-1/PD-L1), chemotherapy, and molecular targeted drugs.

In this article, we will focus on the combined use of molecular targeted drugs with immunotherapy, that could possibly reverse immunosuppression and augment anti-tumor T cell responses.

## Mechanisms of Immunosuppression in Cancer Patients

Cancer cells, more specifically oncogene activation and subsequent signal activation in cancer cells, trigger multiple immunosuppressive cascades. These immunosuppressive cascades involve various immunosuppressive molecules such as TGF-β, IL-10, IL-6, VEGF, PD-L1, COX2, and IDO/TDO as well as immunosuppressive cells such as tolerogenic DCs, myeloid-derived suppressor cells (MDSCs), and regulatory T cells (Tregs). Ultimately, cancer cells generate immunosuppressive microenvironments in tumor and sentinel lymph nodes (Yaguchi et al., 2011). For example, an over production of TGF-β in tumor microenvironment resulted in accumulation of MDSCs, M2 macrophages and Tregs, and impairment of DC functions in tumor tissues and sentinel lymph nodes. We have shown that TGF-β-induced-Snail not only induces metastasis-causing epithelial-to-mesenchymal transition (EMT) of cancer cells but also enhances production of immunosuppressive cytokines and chemokines, including TGF-β, IL-10, CCL2, and TSP-1 (Kudo-Saito et al., 2009), which further promotes metastasis. These cytokines impair DC function, induce Tregs, and finally inhibit induction of anti-tumor T cells. CCL2 produced by cancer cells recruits MDSCs into tumor and CCL22 produced by M2 macrophages recruits CCR4^+^ Tregs and Th2 cells into tumor and sentinel lymph nodes (Kudo-Saito et al., 2009, 2013; Tsujikawa et al., 2013). Therefore, TGF-β production in tumor microenvironment by either cancer cells or infiltrated immune cells triggers multiple immunosuppressive cascades involving various immunosuppressive cytokines, chemokines, and immune cells. It has been reported that inhibition of TGF-β signaling by injection of plasmid DNA containing TGF-β type II receptor cDNA near the tumor sites enhanced tumor antigen-specific T cells accompanied by decrease of Tregs through blockade of TGF-β signaling (Fujita et al., 2009). Therefore, blockade of the TGF-β dependent immunosuppressive cascade at either upstream signaling for TGF-β production, TGF-β itself, or its downstream events such as Treg induction may restore immunocompetence of cancer patients.

## Signal Inhibitors may Augment Anti-Tumor Immune Responses

To effectively reverse immunosuppressive condition in cancer patients, which molecules or cells should be targeted in the immunosuppressive cascades? Where should they be blocked, upstream, or downstream? Blockade of downstream immunosuppressive molecules, such as CTLA-4 and PD-1/PD-L1, was recently shown to be effective in augmenting anti-tumor immune responses in clinical trials (Hodi et al., 2010; Topalian et al., 2012). Targeting downstream immunosuppressive molecules (e.g., TGF-β, IL-10, IL-6, VEGF, CTLA-4, PD-1, PD-L1, IDO/TDO, Cox2) and cells (e.g., MDSCs and Treg) with antibodies or small molecule inhibitors may have specific and efficient inhibitory activity against immunosuppressive cascades. However, inhibition of one molecule or one cell type may not be sufficient to reverse caner immunosuppression in patients.

**Figure 2:**
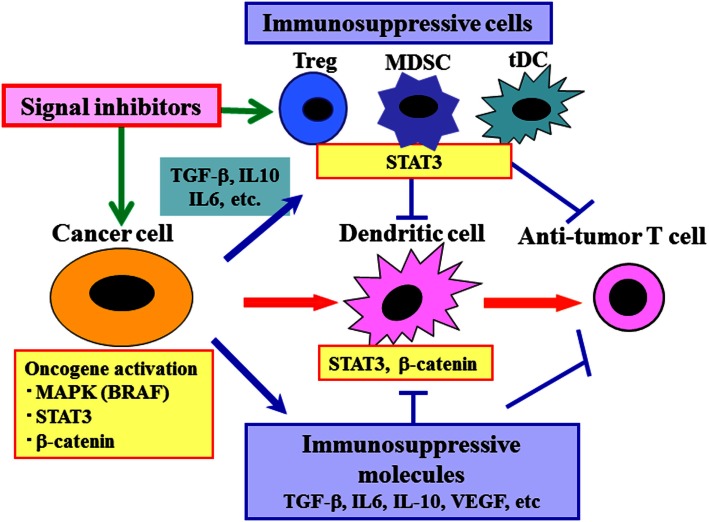
**Reversal of cancer-induced immunosuppression by targeting both cancer cells and immune cells using molecular targeted drugs**. Cancer cells not only trigger anti-tumor immune responses but also induce various immunosuppressive molecules and cells through oncogene and signaling activation, leading to impaired anti-tumor immune responses. Molecular targeted drugs including various signal inhibitors may be useful for augmentation of anti-tumor immune responses by acting on both cancer cells and various immune cells such as DC, MDSC, and Treg.

In order to reverse immunosuppression in tumor-bearing hosts, we have evaluated signal inhibition at upstream molecules, such as BRAF-MAPK, STAT3, and Wnt/β-catenin (Sumimoto et al., 2006; Iwata-Kajihara et al., 2011; Yaguchi et al., 2012) (Figure 2). Targeting a constitutively activated signaling in cancer cells will not only inhibit multiple downstream immunosuppressive events simultaneously but also suppress multiple intrinsic malignant features of cancer cells, such as proliferation, survival, and invasion. The destruction of cancer cells may result in release of various endogenous tumor antigens and contribute to induction of anti-tumor immune response, and subsequent decrease of tumor burden decreases total immunosuppressive activity. In developing molecular targeted therapy, the idea of personalized treatment strategy is crucial. This is because the contribution of target signaling molecules in immunosuppression may be different even among patients with same type of cancer. Another factor to consider is that signal inhibitors sometimes have direct effects on immune cells, including activation of immune cells (e.g., DC) and inhibition of various immunosuppressive cells (e.g., Treg, MDSC) (Iwata-Kajihara et al., 2011; Oosterhoff et al., 2012). A combination of both upstream and downstream blockade is also an attractive strategy. For instance, administration of signal inhibitors (e.g., BRAF inhibitor) and blockade of antibodies against major immuosuppressive molecules (e.g., TGF-β, PD-1/PD-L1, CTLA-4) may be effective. However, it should be noted that such upstream blockade may affect various normal cells and cause adverse effects, including suppression of anti-tumor immune response. Therefore, a careful evaluation of total *in vivo* activity of these signal inhibitors is needed in both animal tumor models and clinical trials.

### MAPK signaling inhibitors

A common mutation of BRAF (V600E), a molecule in MAPK signal pathway, was identified by systematic DNA sequencing of signaling molecules in human melanoma cells (Davies et al., 2002). We have evaluated the role of mutant BRAF (V600E) in human melanoma cells by using mutant BRAF (V600E)-specific lentiviral shRNAs, and found that BRAF mutation was involved in enhanced cell proliferation and invasion (Sumimoto et al., 2004, 2005). We also found that inhibition of MAPK signaling pathway in human melanoma cells by genetic depletion of mutant BRAF or specific inhibitors reduced production of multiple immunosuppressive cytokines such as IL-6, IL-10, and VEGF, in most cases without affecting cell viability (Sumimoto et al., 2006). These cytokines suppress DCs’ ability to stimulate T cells through decreased production of IL-12 and TNF-α and increased production of IL-10 by DCs. Treatment of melanoma cells with BRAF (V600E)-specific shRNA or MEK inhibitors resulted in decreased immunosuppressive activity of melanoma cells on DCs, suggesting that MAPK signaling pathway in cancer is associated with impaired DC function in melanoma patients. MEK inhibitors were reported to increase susceptibility of melanoma cells to CTL lysis partly due to increased expression of melanosomal antigens such as MART-1/melan-A and gp100 (Kono et al., 2006; Boni et al., 2010). These results indicate that the BRAF-MAPK axis is important not only in classical malignant features such as cancer cell proliferation and invasion, but also in immunosuppression and immunoresistance. “Avoiding immune destruction” has recently been recognized as one of the “the hallmarks of cancer” (Hanahan and Weinberg, 2011).

The BRAF-MAPK axis may be a common attractive target for melanoma treatment, including immunotherapy. However, MAPK signaling pathway is also important for normal cell functions, such as T cell proliferation. Thus, administration of MAPK inhibitors may also suppress desirable anti-tumor T cell responses. Recently, two BRAF inhibitors that preferentially inhibit mutant BRAF in cancer cells have been developed, and their administration resulted in regression of melanoma in clinical trials (Chapman et al., 2011). These mutant BRAF-selective inhibitors can be particularly useful in combination with immunotherapies for melanoma. Melanoma cell death induced by BRAF inhibitors may lead to release of multiple endogenous tumor antigens including mutated antigens unique to each patient (Melanoma is known to have more frequent mutations than other cancers probably due to UV irradiation). This results in subsequent induction of autologous tumor-specific T cells. Decreased production of multiple immunosuppressive cytokines along with decreased number of melanoma cells may result in simultaneous inhibition of multiple immunosuppressive cascades, and reduce total immunosuppressive activity of melanoma without suppressing anti-tumor T cell expansion. Increased expression of melanoma antigens leads to enhanced susceptibility of cancer cells to CTL lysis (Kono et al., 2006; Boni et al., 2010). Suppression of melanoma cell proliferation and invasion may also enhance total anti-tumor activity of mutant BRAF inhibitors. In fact, it has recently been reported that administration of the mutant BRAF inhibitors alone resulted in the increased infiltration of granzyme positive CD8^+^ T cells in tumors without inhibiting general immune responses, which was correlated with tumor reduction and necrosis (Wilmott et al., 2011; Hong et al., 2012). In a recent study, mutant BRAF-selective inhibitor and anti-CTLA-4 mAb were used in combination to treat transgenic mice with mutant BRAF and PTEN deletion that spontaneously developed melanoma. Despite their expectation, the combined therapy did not show enhanced anti-tumor effects compared with the treatment with either inhibitor or antibody alone. However, in B16 melanoma model using non-transgenic mice, the anti-CTLA-4 mAb augmented the effects of cancer vaccine (Hooijkaas et al., 2012). Further analysis revealed that BRAF inhibitor did not cause cell death in melanoma of transgenic mouse model, suggesting that *in situ* destruction of cancer cells is an essential step in the enhancement of anti-tumor T cell responses. The mutant BRAF inhibitors may also be useful for treating other cancers that are BRAF mutation positive, such as colon cancer, lung cancer, and thyroid cancer. Although MEK inhibitor is known to suppress proliferation of melanoma with either NRAS or BRAF mutation, it remains to be evaluated whether the inhibitor also has immunological effects, such as stimulating or suppressing activity on anti-tumor T cells (Flaherty et al., 2012).

### JAK/STAT3 signaling inhibitors

STAT3 is frequently activated in various human cancers including melanoma. Similar to the RAS/BRAF/MAPK signaling activation, down-regulation of STAT3 by lentiviral shRNA in STAT3-activated melanoma resulted in inhibition of multiple immunosuppressive cytokines, including IL-6, IL-10, and VEGF, indicating that STAT3 inhibitors may also be useful for immunotherapy (Sumimoto et al., 2006). These suppressive cytokines subsequently activate STAT3 in various immune cells including DCs, MDSCs, and Tregs, and render them immunosuppressive. For example, these cytokines generated low IL-12- and high IL-10-producing human DCs with reduced T cell stimulatory activity. DCs obtained from myeloid-specific STAT3-conditional knockout mice were found to be affected less by cancer-derived immunosuppressive factors (Iwata-Kajihara et al., 2011). In addition, these STAT3-depleted DCs produced high and sustained level of IL-12 possibly due to the involvement of STAT3 in a negative feedback mechanism of DC activation via IL-10. These STAT3-depleted DCs have higher T cell stimulatory activity than wild type DCs. When STAT3-depleted DCs were injected into immunosuppressive tumor microenvironment, stronger anti-tumor effects than wild type DCs were observed along with induction of stronger IFN-γ producing Th1 and CTL (Iwata-Kajihara et al., 2011). It has been reported that STAT3 is also involved in expansion of MDSCs (Wu et al., 2011), activation of CD14^+^HLA-DR^negative/low^ MDSCs in blood of cancer patients (Poschke et al., 2010), expression of immunosuppressive arginase-1 in human MDSCs (Vasquez-Dunddel et al., 2013), survival of Tregs (Pallandre et al., 2007), and anti-tumor activity of CD8^+^ T cells (Kujawski et al., 2010). These reports suggest that constitutive activation of STAT3 in cancer cells triggers induction of various immunosuppressive immune cells. STAT3 inhibitors are currently being evaluated in clinical trials such as NCT00955812. In murine tumor model, STAT3 inhibitors have been shown to augment anti-tumor immunity (Kortylewski et al., 2005; Yu et al., 2007; Lee et al., 2011). It was recently reported that STAT3 inhibitors also restored drug sensitivity of melanoma cells which had acquired resistance to BRAF inhibitors (Liu et al., 2013). Therefore, STAT3 inhibitors may be useful for reversal of cancer-induced immunosuppression through acting on both cancer cells and various immune cells.

Besides direct inhibition of STAT3, inhibitors of the molecules regulating STAT3 activation may also be effective for the reversal of cancer-induced immunosuppression. An inhibitor of JAKs, upstream molecules of STAT3, was reported to augment anti-tumor effects in combination with immunotherapies such as IL-12 administration (Burdelya et al., 2002). In patients with renal cell cancer (RCC), administration of a multikinase inhibitor Sunitinib capable of suppressing downstream STAT3 signaling resulted in decrease of MDSCs and Tregs along with increase of IFN-γ producing T cells (Ko et al., 2009; Ozao-Choy et al., 2009; Xin et al., 2009). Another multikinase inhibitor Dasatinib, which also inhibit downstream STAT3, increased response rate of the patients with Ph1^+^ leukemia (CML and ALL) accompanied by LGL lymphocytosis and autoimmune like syndrome such as pleuritis and colitis (Mustjoki et al., 2009; Jalkanen et al., 2010), suggesting that Dasatinib has immunostimulatory activity partly through STAT3 inhibition. Therefore, various ways of STAT3 signal inhibition may be applicable in combination with various immunotherapies.

### β-catenin-signaling inhibitors

In some human cancers including colon cancer, liver cancer, and melanoma, activation of β-catenin pathway (suggested by nuclear staining of β-catenin) is observed. We found that β-catenin directly promote transcription of immunosuppressive cytokine IL-10 in human melanoma (Yaguchi et al., 2012), and protein expression of β-catenin was correlated with expression of IL-10 when evaluated by immunohistochemical analysis of melanoma tissues samples. Culture supernatant of human melanoma cells with accumulated β-catenin-induced high IL-10- and low IL-12-producing DCs in an IL-10 dependent manner. These DCs possessed low T cell stimulatory activity *in vitro*, and induced FOXP3^+^ immunosuppressive Treg cells. The melanoma derived factors also inhibited the effector function of melanoma-specific CTLs in a β-catenin-dependent, but interestingly IL-10-independent manner, indicating that other immunosuppressive molecules are also involved in the β-catenin-induced immunosuppression. Melanoma cells pretreated with β-catenin-specific shRNA had reduced immunosuppressive activities on both DC and T cells.

When β-catenin-activated human melanoma cell lines were implanted in immunodeficient mice, human IL-10 in mouse serum was increased, and function of mouse DCs in spleens and tumors were impaired for T cell stimulatory activity probably due to increased human IL-10 which is capable of affecting mouse DCs (Yaguchi et al., 2012). Systemic administration of a β-catenin inhibitor restored T cell stimulatory function of the mouse splenic DCs along with decrease of human IL-10 in serum. β-catenin was also reported to be involved in generation of regulatory DC (Fu and Jiang, 2010; Manicassamy et al., 2010a) and survival of Treg (Ding et al., 2008). In addition, β-catenin inhibitor had a direct ability on DC to augment their T cell stimulatory activity partly due to decreased IL-10 production by DC (Manicassamy et al., 2010b). Therefore, β-catenin inhibitors may also be useful for reversal of cancer-induced immunosuppression by acting on both cancer and immune cells.

## Concluding Remarks

As discussed in this article, altered activation of various oncogenes and signaling in both cancer cells and immune cells can be an attractive target to reverse immunosuppressive conditions in tumor-associated microenvironments of cancer patients. Signal inhibitors may augment current cancer immunotherapy, in addition to its possible direct anti-tumor effects through inhibition of cancer cell proliferation and invasion. However, its total *in vivo* activity should be carefully evaluated because it may also cause various adverse effects, including possible inhibition of anti-tumor immune responses. In this regard, mutated-molecule-specific inhibition such as that of the mutant BRAF-selective inhibitors is one of the promising strategies. Activation of STAT3 appears to shift immune response toward cancer’s advantage, thus, its inhibition is attractive for possible improvement of anti-tumor immune responses. Altogether, combination therapy using molecular targeted drugs and various immunotherapies such as cancer vaccines and check point blockade is a promising strategy to treat cancer patients. Future clinical trials may demonstrate the proof of concept of this strategy.

However, there are several obstacles to overcome before the benefits of combination therapy can reach the patients. One such obstacle is scientific. Although quite a few signal inhibitors, immunotherapies, and combined therapies have shown promising results in experimental settings, mouse model, and human are different. A successful treatment in mouse models may not work in patients. Therefore, for the selection of appropriate molecular targets and inhibition methods, further understanding of human cancer immunopathology is deeply essential and urgently desired. Another obstacle is a pragmatic one, which may arise when individual therapies in a combination therapy are developed and/or owned by different companies. The issues of company regulations, patents, and logistics could become a barrier between research and clinical translation. The core idea of combination therapy is that by using multiple already-available therapies, cancer patients are able to gain greater-than-sum benefits. Therefore, it is crucial that institutions and companies to look beyond self-interests and work together to reach a common goal. Academic institution may mediate the cooperation between companies and provided combination therapies to patients.

## Conflict of Interest Statement

The authors declare that the research was conducted in the absence of any commercial or financial relationships that could be construed as a potential conflict of interest.
